# Food for thought: why does the medical community struggle with research about nutritional therapy in the acute care setting?

**DOI:** 10.1186/s12916-017-0812-x

**Published:** 2017-02-24

**Authors:** Philipp Schuetz

**Affiliations:** Department of Endocrinology, Diabetes and Clinical Nutrition, University Department of Internal Medicine, Kantonsspital Aarau and Medical Faculty, University of Basel Switzerland, Kantonsspital Aarau, Tellstrasse, CH-5001 Aarau, Switzerland

**Keywords:** Nutrition, Acute illness, Malnutrition, Inflammation

## Abstract

Although clinical nutrition is a frequently used intervention in inpatient care, high quality trials proving its effectiveness and safety when used in the acutely-ill polymorbid medical inpatient population are largely lacking. From an evolutionary perspective, illness-related low appetite is protective and part of the host response to improve recovery from disease. Large and well performed trials in the intensive care setting have shown deleterious effects of (parenteral) feeding strategies aiming at higher caloric intakes compared to lower intakes, raising the question of whether feeding per se may be simply maladaptive in acute severe illness. Outside critical care, similar large-scale studies are lacking with basic clinical questions regarding the optimal amount/composition of nutrition and best patient selection remaining largely unanswered. Also, the interplay of nutritional interventions and its influence on the microbiome remains largely unclear. Given the magnitude of morbidity caused by malnutrition and the high number of affected patients, it is surprising how little the medical community has invested in better understanding ways to improve this condition. It is now time to perform high-quality trials to better understand how to best deal with this condition in the acute care setting. Such trials will allow change from a one-size-fits-all approach, to more evidence-based, personalized nutritional interventions, ultimately improving patient outcomes. While there is ongoing discussion about definition of malnutrition, we should rather focus on the identification of patients who do or do not benefit from nutritional interventions.

## Background

Nutritional research experienced a very promising start in 1747 with James Lind conducting the first ever randomized controlled trial comparing six different treatments for 12 sailors with scurvy [1]. Hippocrates, himself, had great hopes for nutritional interventions to cure disease (“*Let food be thy medicine and medicine be thy food*”). Today, however, there are few rigorously conducted trials of nutritional interventions that assess their impact on mortality, morbidity, or other patient-centered outcomes. This is particularly true for the acute, non-critical medical care inpatient setting, where there is an important lack of studies to guide clinicians in the optimal use of nutrition in an “evidence-based” manner and help us understand the potential of nutrition to cure disease, or at least to improve patient outcomes [2].

## Nutritional therapy

Regarding the question of nutrition for primary prevention of disease, several large randomized trials have demonstrated impact on mortality and morbidity. For example, the PREDIMED trial showed the positive effects of a Mediterranean diet on cardiovascular and metabolic disease [3]. Additionally, several recent large trials have had an important impact on today’s management of patients in the critical care setting [4–6]. Within this population, if insufficient nutritional support is provided, patients will accumulate a large energy deficit that may contribute to lean-tissue wasting, which is associated with adverse outcomes. Yet, surprisingly, recent evidence has pointed to detrimental effects of feeding strategies in the setting of critical illness, with possible negative effects on cell recycling (autophagy) and an increase in morbidity and mortality [4]. Further, worse outcomes due to refeeding associated with early feeding have also been reported [7]. These trials challenge our understanding of “physiological nutrition” in the context of severe illness. Thus, recent recommendations based on these trials suggest a hypocaloric macronutrient intake during the first week of critical illness and a continuous increase once the patient’s condition has stabilized [5].

Evidence from critical care studies showing harmful effects when clinical nutrition is used [4, 6] highlights the unanswered questions regarding whether acutely ill, malnourished inpatients benefit at all from nutritional support or, conversely, whether loss of appetite should be viewed as a protective physiological response. In fact, feeding in acute severe illness may be simply maladaptive. While observational research has reported strong associations of malnutrition and adverse outcomes, and suggested benefit from nutritional intervention in the medical inpatient setting, only randomized trials can prove that such interventions truly work. A recent meta-analysis focusing on randomized trials in the medical inpatient setting that compared patients receiving nutritional interventions with patients not receiving such an intervention found that nutritional therapy increases caloric and protein intake, as well as weight [8]. Regarding clinical outcomes, unplanned hospital readmission rates were significantly improved by the intervention, as well as lengths of stay in the subgroup of patients with established malnutrition. However, no effects were found on functional outcomes, infection risk, or mortality – arguably the most important outcomes. Importantly, this meta-analysis also pointed out the low quality of most of the 22 included trials (with a total of 3736 participants), with high heterogeneity across trials and an overall unclear risk of bias. Thus, the lack of significant results regarding mortality and morbidity may be due to methodological issues and low statistical power, and not due to a lack of effectiveness. Indeed, since the publication of this meta-analysis, the large-scale, multicenter, placebo-controlled, interventional NOURISH trial including older malnourished adults hospitalized for congestive heart failure, acute myocardial infarction, pneumonia, or chronic obstructive pulmonary disease was published, that found a significant lower mortality in patients when a high-protein oral nutritional supplement containing beta-hydroxy-beta-methylbutyrate was added to standard-of-nutritional-care [9].

Remarkably, therefore, basic questions such as, “What is the optimal amount of calories and protein for medical inpatients?”; “Which medical inpatients have the most benefit from nutritional interventions?”; and “What type of nutrition (if any) is beneficial for the medical inpatient?” remain largely unanswered [10]. Further, from an evolutionary perspective, it remains unclear whether nutritional interventions should indeed be used in patients with a functional gastrointestinal tract and low appetite. The loss of appetite associated with severe disease may in fact be a protective, physiological response and not a therapeutic target that can be positively influenced by nutritional interventions [11]. Instead of continuing to use nutritional therapy in the acute care setting based on a “gut-feeling” and on results from mainly observational and smaller randomized, hypothesis-generating studies, we should now take a step back and conduct high quality, randomized controlled trials that will aid the understanding of what, if anything, constitutes evidence-based nutritional therapy in specific hospitalized populations [2]. While there is ongoing discussion about the best definitions of malnutrition [12], we should rather focus on the identification of patients who do or do not benefit from nutritional interventions. Specific biomarkers may be helpful to select populations in which nutritional interventions will have the best effects and those which would experience negative outcomes [13].

In the acute care medical setting – outside critical care – malnutrition remains an increasing problem, affecting as much as 30–50% of patients [14]. Undoubtedly, there is a strong association between malnutrition and adverse clinical outcomes, including higher mortality and morbidity and increased lengths of hospital stay [14]. These associations tempt us to believe that improvement in nutritional status by the use of nutritional interventions also leads to improvements in patient outcomes. However, similar findings have been reported for nutritional factors, such as vitamin D, where associations suggested harmful effects, but intervention research failed to find benefit [15, 16]. Still, from a pragmatic standpoint, despite having few rigorously designed nutritional intervention trials in acute care patients, there remain several reasons to support the current approach of systematically screening inpatients for malnutrition and commencing nutritional therapy in those identified. First, an inadequate dietary intake leads to dysfunction of the immune system and mucosal damage in the gut, which increases the risk for diarrhea, malabsorption, bacterial invasion (translocation), and infections [11]. Second, malnutrition is associated with adverse metabolic consequences, such as catabolism and muscle wasting, and epidemiological studies from various countries and healthcare settings have shown strong associations of malnutrition and adverse patient outcomes. Third, a retrospective analysis of controlled trials found positive effects of nutritional support if patients had either impaired nutritional status or an increase in nutritional requirements resulting from high severity disease, or both [17]. Fourth, the above mentioned meta-analysis [8], as well as the very recent NOURISH trial [9], found some positive effects of nutritional support on readmission and mortality in medical inpatients.

## Conclusions

Despite strong associations between malnutrition and adverse outcomes, causal inferences remain largely unproven. Additionally, whether provision of nutritional therapy in the acute phase of a medical illness has the potential to reverse the adverse effects associated with malnutrition in the non-critical care inpatient setting remains unclear. Given the magnitude of morbidity caused by malnutrition and the high number of patients affected by this condition, it is surprising how little the medical community has invested in recent years to better understand ways to improve this condition in an “evidence-based” manner. Although the use of nutritional therapy is a common intervention in medical inpatients, there is no strong current scientific evidence of its efficacy or standard algorithms for its use in medical inpatients. This may be explained by difficulties in standardization, blinding, and patient compliance in nutritional trials, and insufficient industry-funding due to low product margins, among other issues [18]. Still, considering recent high-quality evidence from critical care demonstrating the negative effects of nutritional therapy, a reappraisal of how nutritional support should be implemented in the medical inpatient setting is now required. The selection, timing, dose and feasibility of nutritional treatment should be evaluated as carefully as with any other therapeutic interventions, with the aim of maximizing efficacy and minimizing side effects and costs. Further, trials should investigate the effects of nutritional interventions on the microbiome, which may play a key role in this issue. Instead of pragmatically using nutritional interventions based on few rigorous randomized trials, we should return to James Lind’s research of 1747 and perform the large and well conducted trials needed to truly understand the potential of nutritional therapy to positively influence recovery from disease in the medical inpatient population.
